# The reliability, validity and clinical utility of the Clinical Outcomes in Routine Evaluation – ten-item version (CORE-10) in post-acute patients with stroke

**DOI:** 10.1177/02692155241236602

**Published:** 2024-03-05

**Authors:** Tom Steverson, Joseph Marsden, Joshua Blake

**Affiliations:** 1 6106 University of East Anglia, UK; 2 232261 Norfolk Community Health and Care NHS Trust, UK

**Keywords:** Stroke, Clinical Outcomes in Routine Evaluation – ten-item version (CORE-10)‌, mood screening, mood assessment‌, reliability, validity

## Abstract

**Objective:**

To explore the validity, reliability, and clinical utility of the Clinical Outcomes in Routine Evaluation – ten-item version (CORE-10: a ten-item questionnaire designed to measure psychological distress) in a stroke inpatient sample and calculate reliable and clinically significant change scores.

**Setting:**

A post-acute stroke rehabilitation ward in the East of England.

**Participants:**

A total of 53 patients with stroke, capable of completing the CORE-10 as part of their routine clinical assessment. Exclusion criteria included moderate to severe aphasia and/or alexia.

**Main measures:**

Alongside the CORE-10, the Patient Health Questionnaire – 9, the Hospital Anxiety and Depression Scale, the Centre for Epidemiological Studies-Depression Scale, and the Beck Depression Inventory Second Edition were used as concurrent measures.

**Results:**

To assess reliability, the internal consistency and test–retest reliability of the CORE-10 were calculated. The average number of days between CORE-10 test–retest administrations was 2.84 (*SD* = 3.12, *Mdn* = 1). Concurrent validity was assessed by examining correlations between the CORE-10 and comparable measures, and clinical utility was assessed using the criteria of Burton and Tyson (2015). The internal consistency (Cronbach’s alpha) for the CORE-10 was .80, and test–retest reliability interclass correlation coefficient was .81. Total score correlations between the CORE-10 and concurrent measures ranged from *r* = .49 to *r* = .89. The CORE-10 achieved the maximum score (i.e. 6/6) on criteria for clinical utility. Calculations demonstrated a reliable change index of nine points and a clinically significant change cut point of 12 on the CORE-10. Percentiles for CORE-10 total scores are reported.

**Conclusions:**

This study provides preliminary support for the CORE-10 as a valid and reliable measure that has clinical utility for screening distress in inpatients with stroke.

## Introduction

United Kingdom national guidelines recommend routine assessment of mood difficulties after stroke.^1,2^ More detailed guidance on the types of mood assessment to use is outlined by the Sentinel Stroke National Audit Programme, which audits data on stroke service mood screening rates in the United Kingdom. The Sentinel Stroke National Audit Programme considers an acceptable mood assessment to be one that has published/peer reviewed evidence of validity in clinical use, preferably in stroke, and its use has been approved by an appropriate body (e.g. the clinical service governance group) of the trust in which it is used.^3^ The Sentinel Stroke National Audit Programme^4^ provides specific examples of recommended validated tools. However, one measure that is not featured in the recommendations is the Clinical Outcomes in Routine Evaluation – ten-item version (CORE-10),^5^ which may have several advantages over other measures. For example, in the context of routine mood screening, a brief questionnaire containing only 10 items may be desirable. The CORE-10 is also freely available, incurring no costs or specialist training to use, and demonstrates good psychometric properties in clinical and non-clinical populations.^5^ Equally, the CORE-10 is considered a measure of “psychological distress”^5^ rather than anxiety and depression in isolation. A focus on distress may be preferable for routine mood screening after a stroke; as the UK and Ireland National Clinical Guidelines for Stroke state “many people with stroke are troubled by distress that does not meet diagnostic criteria for depression and anxiety … Depression and anxiety are closely linked and may be part of a single emotional response to stroke”.^1^ Moreover, unlike some of the questionnaires endorsed by SSNAP, the CORE-10 also includes a question about suicide plans, which is important as the risk of suicide after a stroke is increased.^6^

Despite the apparent advantages of the CORE-10 in stroke rehabilitation, it is yet to be validated in this population. This study therefore aimed to address the following questions in a post-acute, stroke inpatient sample:

1. What is the validity and reliability of the CORE-10?
2. What is the clinical utility of the CORE-10?
3. What is the reliable change index and clinically significant change cutting score for the CORE-10?

## Method

The study used a cross-sectional design. A total of 53 participants were recruited from a 24-bedded, post-acute rehabilitation ward in the East of England. Patients arrived on the ward for a period of rehabilitation, typically following discharge from an acute setting. Data from the Sentinel Stroke National Audit Programme showed that across the five years between April 2018 and March 2023, the average annual length of stay on the ward ranged from 51.8 to 78.9 days. Participants were included if they were judged to be able to complete the CORE-10 as part of their routine clinical assessment. Exclusion criteria included moderate to severe aphasia/alexia that would compromise the ability to participate and/or lacking the capacity to consent to take part in research. The sample had a mean age of 72 years on the date of entering the study, and 51% were female. The type of stroke^7^ was available for 45 (85%) of participants, which was recorded on their discharge letter from the acute hospital. Of the 45, 10 (22%) had total anterior circulation syndrome, 17 (38%) had partial anterior circulation syndrome, four (9%) had posterior circulation syndrome, and 14 (31%) had lacunar syndrome.

Potential participants who met the inclusion/exclusion criteria were approached by a member of the ward staff and were asked if they would like to find out more about being a participant in the study. Participants expressing interest were approached by a member of the research team, wherein further information about the study was provided, and written informed consent was obtained from those who agreed to participate. The number of participants who were approached who refused to participate was not recorded. If consent was given, a suitable time was arranged to meet and complete study consent forms and mood questionnaires. All study measures were administered in a single session with each session taking approximately 45 minutes. A follow-up CORE-10 was completed within seven days to enable calculation of test–retest reliability. Questionnaires were administered in a random order to mitigate the influence of order effects. The measures were administered by authors TS (clinical psychologist) and JM (assistant psychologist) who, at the time of the study, were working clinically on the ward where the study took place. Participants were given the option of completing the questionnaires either at their bedside or in a private room. To make responding easier for those with mild cognitive or communication difficulties, all participants were presented with the response options for each measure printed in bold, capital letters, font size 36, and in descending order on a horizontal, A4 laminated sheet.

Ethical approval for the study was granted by the Office for Research Ethics Committees Northern Ireland and the NHS Health Research Authority on 18 January 2021 and 17 February 2021, respectively (Integrated Research Application System Project Identification Number: 278081).

Data were analysed using Statistical Package for the Social Sciences (Version 25) and R version 4.11. Reliability was assessed via Cronbach's alpha and test–retest reliability via an interclass correlation coefficient two-way mixed model with absolute agreement. Guidelines on the optimal time difference between the initial test and the retest emphasise the interval period being long enough to prevent recall but short enough to avoid genuine change occurring.^8^ Whilst some authors have recommended an interval of between two and fourteen days,^9,10^ others have avoided specifying a time period, in place of describing and justifying the rationale for the interval chosen. Given the evidence suggesting that mood is highly changeable within inpatient stroke settings,^11^ a shorter target was deemed more appropriate to minimise the risk of bias from actual mood changes. The time difference between the test and the retest was therefore set to *within* seven days.

Concurrent validity was assessed by comparing the strength of correlations between the CORE-10 total score and comparable measures. Clinical utility was defined according to the criteria of Burton and Tyson,^12^ which use a zero to six scale to rate a measure based on the time to administer and score, initial costs to purchase, additional cost per form and the need for specialist training. Higher scores mean greater clinical utility.

Reliable change refers to the extent to which an individual's change score on a measure exceeds that which would be expected from measurement error alone.^13^ We applied the formula described by Jacobson and Truax^14^ which uses the standard error of measurement of an assessment to calculate the standard error of the difference between change two scores. This is a value that represents the spread of the distribution of change scores that should occur if no genuine change took place. Dividing the difference between an individual's pre and post scores by this standard error of the difference score gives a ‘reliable change score’ for the individual, which can be compared to a critical value of a normal distribution. Jacobson and Traux^14^ suggest that individuals with reliable change scores greater than a critical threshold of 1.96 can be considered to have changed reliably (i.e. it is 95% likely that the observed change is not due to measurement error). This reliable change score can also be multiplied by the standard error of difference to give a ‘reliable change index’, which represents the minimum raw change score required on a measure for the change to be considered reliable.^15^ Indeed, the reliable change score is a score calculated for an individual, whereas the reliable change index is a property of a measure.^15^ Whilst there is debate about whether to use Cronbach’s alpha or test–retest reliability coefficient for calculating the initial standard error of measurement as part of the reliable change score calculation,^11^ we chose Cronbach’s alpha as this reliability estimate is not affected by any genuine changes that may have occurred between administrations.^13^

Jacobson and Traux^14^ also give a definition for clinically significant change which concerns the extent to which an individual's score moves from a ‘dysfunctional’ comparison sample range to a ‘functional’ comparison sample range. This can be calculated in three ways (i.e. a, b, or c) depending upon the comparison data available. Criterion ‘a’ can be used when only comparison data from a ‘dysfunctional’ sample is available. It examines whether an individual's pre to post test score moves at least two standard deviations away from the ‘dysfunctional’ sample's mean, in the direction of improvement on a given measure. Criterion ‘b’ is used when only ‘functional’ comparison data is available and examines whether an individual moves to within two standard deviations of the ‘functional’ sample's mean in the direction of improvement. This criterion requires their starting score to be more than two standard deviations from the mean. Criterion ‘c’ is used when both ‘dysfunctional’ and ‘functional’ data are available. Here, a cut-off point can be calculated,^13^ which differentiates moving from a ‘dysfunctional’ into a ‘functional’ comparison group. We used Jacobson and Traux's criterion ‘c’ with a separate sample of 72 patients’ total CORE-10 scores from a previous service evaluation conducted on the same ward as the current study.^16^ This sample had been specifically referred by staff on the ward to the psychology team for assessment or intervention as part of routine clinical practice due to concerns about their mood (where the CORE-10 was either completed immediately prior to the referral to the psychology team or at the first appointment with the psychologist) and hence was defined as the ‘dysfunctional’ sample, which we refer to henceforth as the ‘clinical’ sample. The 53 participants recruited as part of the current study, as described above, were considered the ‘functional’ sample and described henceforth as the ‘research’ sample.

To estimate the sample size required for the correlational analysis, we consulted Moinester and Gottfried^17^ who outline the sample sizes required for values of *r* within a given 95% confidence interval. Here, we wanted to ensure that the sample size would be large enough for the possible value of *r* at the lower bound 95% confidence interval to be meaningful within the context of the existing literature. Based on previous research, we expected correlations of approximately *r* = .68 between the CORE-10 and concurrent measures in this study as this was the average value from the correlations between the CORE-10 and (a) the Patient Health Questionnaire – 9 (*r *= .56), (b) the Beck Depression Inventory – second edition (*r* = .75 and *r *= .76), and (c) the Beck Anxiety Inventory (*r *= . 65) reported by Barkham et al.^5^ across several mixed samples. We set an acceptable 95% confidence interval to be plus or minus an *r* of .20. Using these intervals, Moinester and Gottfried^17^ outline that a sample size of 36 would be sufficient to obtain an *r* value of .65, with the confidence intervals of .55 to .85. Higher values of *r* (such as our predicted value of .68) would require a lower sample size. Given the correlations in the abovementioned literature, we deemed this sample size and these confidence intervals appropriate and enough to detect at least a medium-to-large effect of *r*.^18,19^ For the reliability analysis, Bujang et al.^20^ give the sample sizes required for different levels of Cronbach's alpha based on the number of items in a questionnaire and null and alternate hypotheses for a Cronbach's alpha value. We set our null and alternative Cronbach's alpha values to be .55 and .75, respectively, as these scores would differentiate “nonacceptable” and “acceptable” alpha values.^21^ Accordingly, a sample size of 53 would be sufficient to test the null hypothesis that the alpha value would be .55 and the alternative hypothesis would be .75, based on a significance level of .05, and power set at 80%.

### Measures

The CORE-10^a,^^5^ is a ten-item, abbreviated version of the CORE-Outcome Measure.^22^ The CORE-10 has demonstrated excellent psychometric properties in primary care mental health contexts^5^ and young people.^23^ The CORE-10 uses a five-point ordinal scale of zero to four, where higher scores indicate more severe distress giving a maximum score of 40.

The Beck Depression Inventory – second edition^24^ is a 21-item measure which has been found to have good reliability and validity in a meta-analysis of 144 studies of mixed samples.^25^ In stroke samples, the Beck Depression Inventory – second edition has been shown to have sensitivity and specificity values of 91% and 30%, and 92% and 71% for detecting depression respectively.^12^

The Center for Epidemiological Studies-Depression Scale^26^ is a 20-item measure which has demonstrated high diagnostic accuracy in various samples.^27^ In stroke samples, the Center for Epidemiological Studies-Depression Scale has been found to have sensitivity and specificity values of 86% and 90%, 60% and 76%, and 73% and 100%, for detecting depression respectively.^12^

The Hospital Anxiety and Depression Scale^28^ is a 14-item measure containing two, seven-item subscales which assess anxiety (Hospital Anxiety Scale) and depression (Hospital Depression Scale), respectively. Because the Hospital Anxiety and Depression Scale was designed for use in medical settings, it has less focus on the somatic aspects of anxiety and depression and leans more on cognitive–affective components.^29^ The Hospital Anxiety and Depression Scale has been investigated comprehensively in stroke samples, and it demonstrated excellent internal consistency.^30^ Studies have varied in their reporting of the optimal cut-off points across stroke samples, with Hospital Anxiety Scale cut-offs ranging from 3/4 to 6/7 and Hospital Depression Scale cut-offs ranging from 2/3 to 7/8.^12^

The Patient Health Questionnaire – 9^31^ is a nine-item depression questionnaire with extensive validity evidence in multiple health and mental health populations.^32^ It has demonstrated adequate internal consistency and inter-rater reliability^33^ and excellent diagnostic accuracy in stroke.^12^

## Results

All 53 participants completed the full set of initial questionnaires, and 50 completed a follow-up CORE-10 to enable the calculation of test–retest reliability. The three participants were lost to follow-up due to discharge from the ward before the retest could be carried out. The median number of days between the stroke and completing the first set of questionnaires was 46 (*M* = 53.8, *SD* = 29.7). All data were complete apart from two items on the Center for Epidemiological Studies-Depression Scale, where separate participants did not have responses to questions four and sixteen. These cases were excluded case wise.

Sample characteristics are outlined in Table 1. The clinical and research samples did not significantly differ with respect to age, *U = 1781.5, Z = −.64, p = .527,* or gender percentage, *X^2^ = .517, p = .472.* CORE-10 scores were significantly higher in the clinical sample than in the research sample, *U = 1024.0, Z = −4.42, p < .001*.

**Table 1.** Sample characteristics.

	Healthy sample	Clinical sample
Age (*SD*)	71.6 (13.19)	69.7 (14.5)
Number of males	26 (49%)	40 (56%)
Number of females	27 (51%)	32 (44%)
Mean total CORE-10 score (*SD*)	9.53 (7.05)	15.86 (8.15)

Significant deviations from a normal distribution were identified by Kolmogorov–Smirnov tests in the research sample on the CORE-10, Hospital Anxiety Scale, and The Beck Depression Inventory – second edition (all *p* < .05). Shapiro–Wilk tests also indicated significant deviation for these measures as well as for the Patient Health Questionnaire – 9 and Center for Epidemiological Studies-Depression Scale *(*all *p* < .05)*.* Only the Hospital Depression Scale and Hospital Anxiety and Depression Scale did not deviate significantly from normality across both tests. A visual inspection of histograms confirmed positive skews for all measures apart from the Hospital Depression Scale and Hospital Anxiety and Depression Scale, which appeared approximately normal. Positive skews might be expected from the present studies’ research sample as the measures used are designed to identify individual differences in clinical populations.^34^ Indeed, the distribution of CORE-10 scores for the clinical sample did not show significant deviations from normality on Shapiro–Wilk and Kolmogorov–Smirnov tests, and by visual inspection the histogram appeared normal. Nonetheless, in testing for homogeneity of variance, the CORE-10 total scores were equal across the clinical and research groups *F*(1, 123) = 1.54, *p* = .216. Taking all the above into account, for the analysis of the research sample, non-parametric statistics were used.

The Cronbach’s alpha coefficient for the CORE-10 was .80 for the research sample. A two-way mixed model with consistency agreement intraclass correlation coefficient gave 95% confidence intervals of .71 to .87. Comparisons with the Cronbach’s alpha coefficient for other measures can be seen in Table 2. The analysis showed that this value rose to .82 if item two (close relationships) was deleted. Deleting other items produced no further increase, and the ten original items of the scale were used in all analyses. The Cronbach’s alpha coefficient of the CORE-10 at the retest timepoint was .76.

For test–retest reliability, the average number of days between CORE-10 administrations was 2.84 (*SD* = 3.12, *Mdn* = 1). A two-way mixed model with absolute agreement indicated an interclass correlation coefficient of .81 (95% CI: (.68, .87)) for the sample of 50 participants with complete data on the CORE-10. A Wilcoxon signed rank test showed no significant differences between CORE-10 total scores between administrations (*Z* = −.929, *p =* .353).

All correlations between the CORE-10 and concurrent measures were large^18,19^ and statistically significant at the *p* < .01 level (see Table 2). The CORE-10 was most strongly associated with the Center for Epidemiological Studies-Depression Scale and Hospital Anxiety and Depression Scale scores, sharing over 50% of variance with each respective measure. Notably, the CORE-10 correlated more strongly with the Hospital Anxiety and Depression Scale score than with either the Hospital Anxiety Scale and Hospital Depression Scale sub scores, supporting the CORE-10 as a general distress measure rather than a measure of anxiety or depression alone.

**Table 2.** Measure descriptives and Spearman correlation matrix of total scores.

	CORE-10	BDI-II	CES-D	HADS A	HADS D	HADS T	PHQ-9
Median	7	13	14	5	7	15	8
Mean (SD)	9.5 (7.0)	13.3 (10.1)	15.1 (10.0)	6.7 (4.5)	7.2 (4.4)	13.5 (8.0)	8.1 (6.1)
Internal consistency	.80	.89	.87	.85	.78	.87	.80
BDI-II	.56						
CES-D	.82	.71					
HADS A	.68	.66	.73				
HADS D	.69	.67	.69	.60			
HADS T	.75	.74	.78	.89	.88		
PHQ-9	.56	.67	.67	.49	.58	.60	

*Note.* All correlations are significant at *p* < .01 (one-tailed). CORE-10, Clinical Outcomes in Routine Evaluation – Ten-Item Version; BDI-II, Beck Depression Inventory – second edition; CES-D,  Center for Epidemiological Studies-Depression; HADS A, Hospital Anxiety Scale; HADS D, Hospital Depression Scale; HADS T, Hospital Anxiety and Depression Scale total score (i.e. sum of HADS A and HADS D), PHQ-9, Patient Health Questionnaire – 9.

All measures used in this study were rated against the criteria developed by Burton and Tyson^12^ for scoring the clinical utility of screening tools for mood difficulties after a stroke in Table 3. The scores for the Beck Depression Inventory – second edition, Center for Epidemiological Studies-Depression Scale, Hospital Anxiety and Depression Scale, and Patient Health Questionnaire – 9 are taken from those reported by Burton and Tyson.^12^ The CORE-10 and Patient Health Questionnaire – 9 achieved maximum scores.

**Table 3.** Clinical utility of measures used in the study.

Criterion	Scoring	CORE-10	BDI-II	CES-D	HADS	PHQ-9
Time to administer and score	≤5 minutes = 2 6–10 minutes = 1 ≥11 minutes = 0	2	1	0	1	2
Initial costs for purchase	Freely available = 2 <£100 = 1 ≥£100 or unavailable = 0	2	0	2	0	2
Additional cost per form	No additional cost = 1 Additional cost or unavailable = 0	1	0	1	0	1
Need for specialist training	No specialist training required = 1 Specialist training required = 0	1	1	1	1	1
Total		6	2	4	2	6

*Note.* Higher scores mean greater clinical utility. CORE-10, Clinical Outcomes in Routine Evaluation – ten-item version; BDI-II, Beck Depression Inventory – second edition; CES-D, Center for Epidemiological Studies-Depression; Hospital Anxiety and Depression Scale; PHQ-9, Patient Health Questionnaire – 9.

Reliable change indices were calculated for the clinical group using the method of Jacobson and Truax,^14^ as described above. As it was not possible to calculate the Cronbach’s alpha or test–retest coefficients from the clinical sample data, the Cronbach’s alpha value from the research group (i.e. .80) was used. The use of a critical value of 1.96 (as used in the original formula to derive a reliable change index that is unlikely to occur more than 5% of the time because of the unreliability of the measure alone) gave a reliable change index of 10.1 (rounded down to 10). The use of a critical value of 1.65 (risking 10% measurement error) gave a reliable change index of 8.5 (rounded up to 9).

A cutting point to determine a clinically significant change was developed by using Jacobson and Truax's ‘c’ formula.^14^ This revealed a cutting score of 12.4. Rounding this figure to 12, 30.2% of the research group received a score of 12 and above, and 30.6% of the clinical group scored 11 and below. This finding was further investigated by calculating the percentile ranks of each CORE-10 total score in both samples. As can be seen in the Appendix, 71.2% of the research sample and 32.4% of the clinical sample obtained CORE-10 total scores of ≥12.

## Discussion

This study aimed to evaluate the psychometric properties of the CORE-10 in a post-acute stroke inpatient sample. The CORE-10 has been extensively validated in other populations, and its focus on general distress suggests potential suitability and utility as a routine post-stroke psychological outcome measure. Here, we found evidence of good internal and external consistency, a maximum clinical utility rating according to Burton and Tyson's criteria,^12^ and acceptable concurrent validity with measures of depression and anxiety. We also report reliable change, clinically significant change, and percentile data to support clinicians in evaluating clinical outcomes and recovery from elevated distress in stroke inpatients.

The Cronbach's alpha value of 0.8, reported here, falls within the “acceptable” range of between 0.70 and 0.95.^21^ and indicates that the CORE-10’s constituent items capture unique variance relating to distress while maintaining consistency and congruence as a measure. Likewise, the test–retest reliability value of 0.8 is within the “good” range of between 0.75 to 0.90.^35^ Together, these findings suggest good measure reliability, which in turn supports the measure's validity and feasibility for establishing reliable and clinically significant change scores.

Concurrent validity correlations were generally high and in keeping with the intercorrelations among other measures, supporting evidence of its validity in stroke inpatients. The exceptionally high agreement between anxiety and depression measures was not expected, or necessarily desirable, given the broader scope of the CORE-10 as a general distress measure and intentional divergence from disorder-specific measures. Such a position was indeed supported by the finding that the CORE-10 correlated more strongly with Hospital Anxiety and Depression Scale score than with either of its subscale scores.

Reported reliable change thresholds suggest that those with clinically elevated distress must improve or deteriorate by at least nine points on the CORE-10 for 90% confidence in reliable clinical change. Changes of 10 points or greater equate to 95% reliable change. The decision to report reliable change indices at both alpha levels is based on suggestions that calculating reliable change indices with lower critical values captures change that is more strongly associated with other outcome variables including satisfaction with treatment, clinician rated post therapy functioning and discharge to a lower level of care.^36^ Indeed, in their original validation of the CORE-10, Barkham et al.,^5^ also report a 90% false positive reliable change rate. Whilst Wise's^37^ suggestion that those showing change associated with 95% confidence may be described as “recovered”, whereas those associated with 90% as “remitted” may be partially useful as a clinical shorthand, we would recommend interpreting these scores in the context of their degree of measurement error.

Nonetheless, it is important to acknowledge that changes of this magnitude (i.e. 10 or nine points) represent sizeable change scores in the context of a measure that is 40 points long. Whilst the reliable change index is a function of the size of the standard deviation in our clinical group (a smaller standard deviation would have produced a lower reliable change index), we would encourage further studies reporting CORE-10 mean and standard deviation scores in samples of people with stroke on inpatient wards who have been referred to psychological therapy services to inform how representative our standard deviation is. This would inform further refinement of the reliable change index reported here.

The final test–retest interval period (i.e. 2.84 days) was at the lower end of the recommended range of two to fourteen days.^9,10^ Whist we based this on a desire to avoid the retest capturing real clinical change, given how changeable mood can be in stroke inpatient settings,^11^ we acknowledge that this makes the influence of bias from recall more likely. Accordingly, we have reported the confidence intervals around the test–retest reliability interclass correlation coefficient and encourage readers to interpret the result in that context.

It is also important to state that some authors have cautioned against the calculation of clinically significant change if distributions are skewed and variances are unequal.^34^ Others have argued that this is only a problem if the data are skewed “severely”.^13^ In this study, whilst CORE-10 scores were positively skewed in the research sample, as is common in “non-clinical”^34^ samples, the variances were equal across the research and clinical samples. Whilst the clinically significant change score of 12 was similar to the cutting scores of 11 for ‘general psychological distress’ and 13 for ‘depression’ in a general population sample^5^ we acknowledge the issues with the distribution of scores and have provided percentile scores for further comparison.

There are several further limitations of this study that must be considered. First, the sample is limited to those on an inpatient rehabilitation ward and those without severe communication difficulties. Accordingly, CORE-10 questions, such as those relating to sleep difficulties, may be answered differently by an inpatient sample thus affecting the ability of this data to be generalised to other stroke populations or contexts.^38^ Second, item-level and longitudinal data were not available for the clinical sample, requiring the assumption, when calculating reliable change index and clinically significant change, that reliability values were equivalent to the non-clinical sample. Finally, we acknowledge that the clinical sample was not derived from a diagnostic interview classification and instead based on clinical concern of referring clinicians. Thus, parameters and percentiles relating to this sample should not be used to make judgements about disorder classification and, instead, they represent the characteristics of clients presenting with elevated distress to non-psychologist clinicians.

Overall, our findings provide preliminary support for the reliability and validity of the CORE-10 in a post-acute stroke inpatient sample. A reliable change index, clinically significant change score, and percentile data are provided, which offer a reference to clinicians in identifying the relative severity of distress compared to those in our sample and an indicator of pre–post changes necessary to be confident of clinical change. The CORE-10 may, therefore, be helpful both as an initial screen for multiple aspects of clinical distress and as an outcome measure for measuring change in non-specific mood difficulties.

**Clinical messages**

- The CORE-10 offers a valid, reliable, and clinically useful way to screen for initial mood difficulties on inpatient stroke rehabilitation wards for those without severe cognitive or language impairment.
- In this sample, an increase/decrease of nine or more points on the CORE-10 is considered a reliable change.
- In this sample, a CORE-10 score of 12 and above indicated general psychological distress.

## Notes

a. A copy of the CORE-10 can be found at https://www.coresystemtrust.org.uk/wp-content/uploads/2020/07/CORE-10-English.pdf.

## Appendix Percentile data for CORE-10 total scores, for each sample

**Table:**

CORE-10 Score	Research sample total	Research male	Research female	Clinical sample total	Clinical male	Clinical female
0	2	4.1	0	0.1	0.1	0
1	3.9	6.1	0.1	2.9	5.2	0
2	7.7	8.1	3.9	4.3	6.5	0.1
3	19.3	24.1	11.6	5.7	7.7	1.1
4	23.1	28.1	15.4	6.4	9	2.2
5	25.1	30.1	19.3	11.3	15.4	3.3
6	38.5	40.1	34.7	14.1	18	6.5
7	53.9	56	50.1	15.5	20.6	7.6
8	54.9	58.1	51.3	17	23.1	8.7
9	55.8	60.1	52.6	22.6	28.3	13
10	63.5	68.1	57.7	25.4	30.8	16.2
11	69.3	76.1	61.6	29.6	33.4	22.6
12	71.2	80.1	63.5	32.4	38.5	24.2
13	75.1	81.4	69.3	38.1	46.2	25.9
14	77	82.7	73.1	42.3	48.8	32.3
15	84.7	84.1	84.7	45.1	51.3	35.5
16	86.6	88.1	85.9	52.2	59	42
17	87.6	89.1	87.2	56.4	64.2	45.2
18	88.5	90.1	88.5	64.8	74.4	51.7
19	90.4	91.1	92.4	71.9	79.5	61.3
20	96.2	96.1	96.2	74.7	82.1	64.6
21	96.4	96.3	96.6	76.1	84.7	65.6
22	96.6	96.6	97.1	77.5	87.2	66.7
23	96.8	96.9	97.5	78.9	88.5	67.8
24	97.1	97.1	97.9	86	92.4	77.5
25	97.3	97.4	98.3	87.4	93.2	80.7
26	97.5	97.7	98.8	90.2	94.1	87.1
27	97.7	97.9	99.2	94.4	97.5	90.4
28	97.9	98.2	99.6	95.1	98.8	90.9
29	98.1	98.4	100	95.8	100	91.4
30	98.4	98.7	100	96.2	100	92
31	98.8	99	100	96.5	100	92.5
32	99.1	99.2	100	96.9	100	93.1
33	99.4	99.5	100	97.2	100	93.6
34	99.7	99.8	100	97.9	100	95.2
35	100	100	100	98.6	100	96.8
>35	100	100	100	100	100	100
